# Correlation analysis and clinical significance of changes in upper thoracic vertebra tilt and clavicle angle pre- and post-operation

**DOI:** 10.3389/fsurg.2024.1264966

**Published:** 2024-02-22

**Authors:** Zhensong Jiang, Haoyu Wang, Ran Cui, Xingpeng Wang, Yunhui Wang, Mingtong Sun, Fushuai Peng, Tao Li, Weimin Zhang, Wen Zhang

**Affiliations:** ^1^ Department of Spine Surgery, Shandong Provincial Hospital Afﬁliated to Shandong First Medical University, Jinan, Shandong, China; ^2^ Center for Reproductive Medicine, Cheeloo College of Medicine, Shandong University, Jinan, Shandong, China

**Keywords:** lateral shoulder balance, upper instrumented vertebra tilt, clavicle angle, scoliosis, correction surgery

## Abstract

The imbalance of the lateral shoulder is reflected by the clavicle angle (CA) in radiology. It remains unclear how to achieve postoperative lateral shoulder balance (LSB) after spinal deformity correction surgery. A retrospective analysis was conducted on AIS patients who underwent surgery by the same spine surgeon at our hospital from 2016 to 2020. A total of 110 patients with spinal deformity were included in the study to verify the correlation between the T1–T5 tilt angle and CA before and after surgery, as well as the relation-ship between the change in T1–T5 tilt angle before and after surgery and the change in CA before and after surgery. By comparing the correlation coefficients, it was found that there may not be a direct relationship between the pre- and postoperative tilt angles of T1–5 and CA, but their changes were closely related to the changes in CA. The change in T1 tilt angle after orthopaedic surgery was significantly correlated with the change in CA, with a correlation coefficient of 0.976, indicating a close relationship between T1 and the clavicle. As the vertebrae moved down, the correlation gradually decreased. In summary, this study suggests that there is a close relationship between T1–T5 and the clavicle and that the change in T1 tilt angle after spinal scoliosis correction surgery is significantly correlated with CA, which decreases as the vertebra moves down.

## 1 Introduction

Adolescent idiopathic scoliosis (AIS) is a three-dimensional spinal deformity characterized by coronal plane curvature, sagittal plane spinal deviation, and transverse plane vertebral rotation. Early-onset scoliosis (EOS), which appears before the age of 10, can be due to congenital vertebral anomalies, neuromuscular diseases, scoliosis-associated syndromes, or idiopathic causes. It can have serious consequences for lung development and significantly reduce the life expectancy compared to adolescent scoliosis (1). For patients with significant spinal deformities, surgical treatment is still considered the only definitive treatment option, with the aim of achieving three-dimensional correction and stable spinal joint fusion to prevent further progression of scoliosis (2). For severe early-onset scoliosis, growth rods are now considered the gold standard for spinal corrective surgery (3, 4). Postoperative shoulder imbalance (PSI) is a significant complication of scoliosis correction surgery. Although many spine surgeons have studied PSI extensively, its incidence remains high, with a reported rate of 25% (5). PSI has two different types: medial shoulder imbalance (MSI) and lateral shoulder imbalance (LSI) (6). MSI is reflected radiographically by the T1 tilt angle (the angle between the horizontal line and a line passing through the superior endplate of T1), first rib angle (FRA, the angle of inclination between lines connecting the top of the first rib), and the degree of curvature of the proximal thorax (PT) (7, 8), while LSI is reflected by the clavicle angle (CA, the angle between a line connecting the highest points of each clavicle and the horizontal line) and the difference in height of the manubrium (CHD, measured by drawing a horizontal line at the upper edge of each manubrium to determine the difference in height between the two sternal notches) (8, 9). Currently, there are reports in the literature indicating that imaging parameters such as T1 tilt angle have good correlation with MSI but poor correlation with LSI and cannot be used as a substitute indicator for intraoperative lateral shoulder balance (LSB) (10). However, our study found that although the correlation between T1 tilt angle and CA was weak preoperatively and postoperatively, there was a significant correlation between changes in T1 tilt angle and changes in CA after corrective surgery. Therefore, the purpose of this study was to investigate the relationship between preoperative and postoperative T1–T5 tilt angles and their changes and CA, providing a new imaging indicator for postoperative shoulder balance after corrective surgery.

## 2 Materials and methods

### 2.1 Patient population

**Figure 1:**
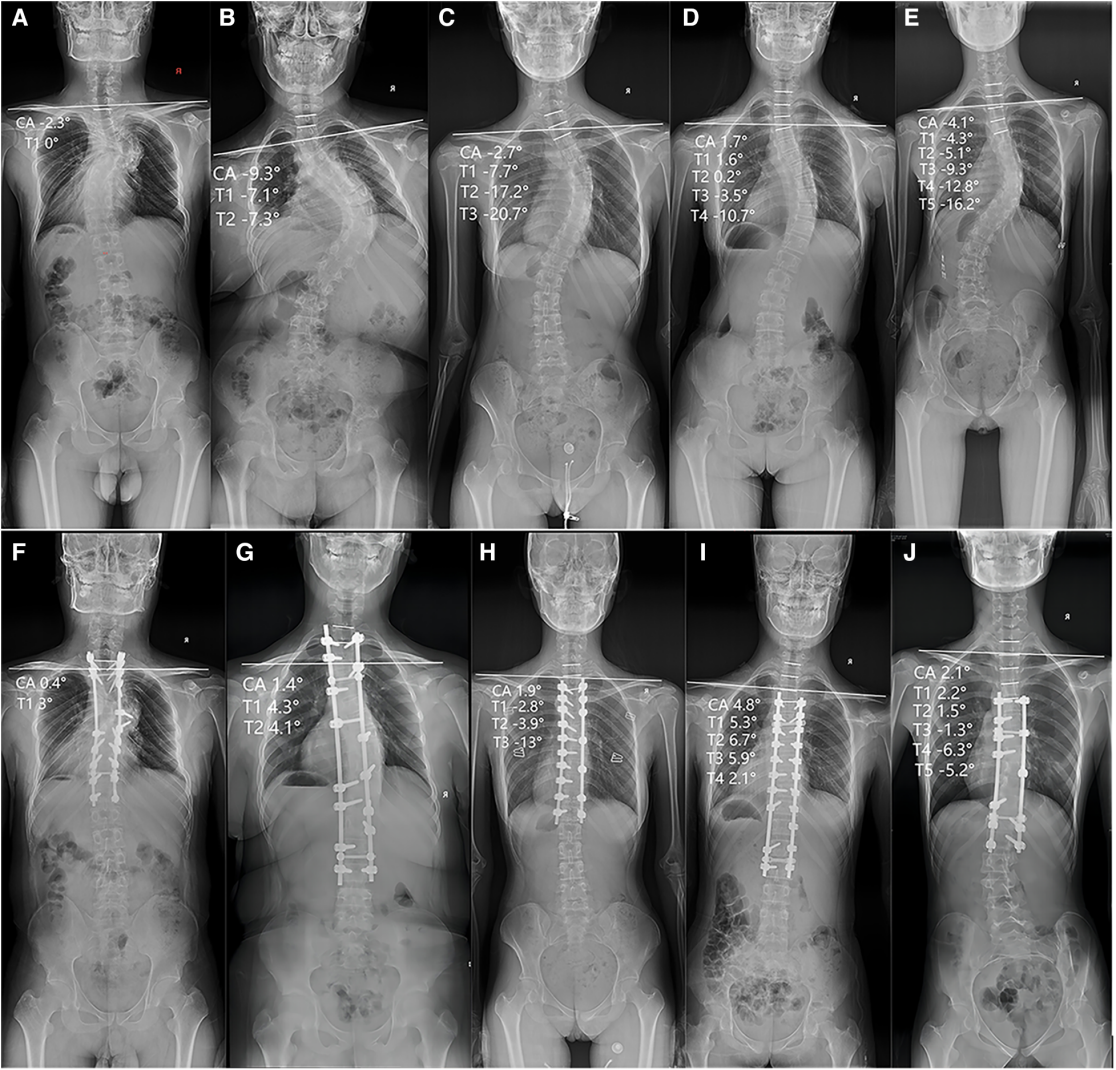
Representative scoliosis cases in the retrospective study and includes the AP radiograph of the entire spine preoperatively and the AP radiograph of the entire spine postoperatively. The (**A**) and (**F**) represent the preoperative and postoperative entire spine radiograph of patients with UIV located in T1, respectively. The (**B**) and (**G**) represent the preoperative and postoperative entire spine radiograph of patients with UIV located in T2, respectively, and the same applies to (**C,H**), (**D,I**) and (**E,J**).

After obtaining approval from the institutional review board, a retrospective review of medical records and spinal x-rays was performed on AIS patients who underwent surgical treatment from 2016 to 2020. We analysed 100 AIS patients and 10 EOS patients from a previous study database, and all corrective surgeries were performed by senior spinal surgeons in our hospital. All x-rays were taken in a standardized manner, with preoperative full-spine anteroposterior (AP) and lateral x-rays obtained and postoperative full-spine standing and lateral x-rays obtained immediately after surgery. Two spinal surgeons who did not participate in the surgery used Surgimap professional measurement tools to measure the preoperative T1–T5 tilt angle, preoperative clavicle angle (CA), postoperative T1–T5 tilt angle, and postoperative CA. Age, gender, and weight were collected. All x-ray measurements were completed by two authors who did not participate in patient treatment. The clavicular angle is defined as the angle between the highest point of the clavicle and the horizontal line. A positive clavicle angle is defined as tilting to the right, and a negative angle is defined as tilting to the left. The T1 tilt angle is defined as the angle between the line drawn along the endplate of T1 and the horizontal line. A positive T1 tilt angle is defined as tilting to the right, and a negative angle is defined as tilting to the left (10, 11). The T2-T5 measurement method is the same as that of T1, as shown in Figure 1. We divided patients with the UIV located in T1–5 into five groups: Group T1, Group T2, Group T3, Group T4 and Group T5. To avoid the impact of surgical internal fixation on the inclination angle of the vertebral body, we only measured the tilt angle at and above the UIV when conducting data statistics. For example, when the UIV is located at T2, we only measure the tilt angle of T1 and T2. When analysing the relationship between T1 tilt and CA, we included T1 tilt and CA data from all groups. When analysing the relationship between T2 tilt and CA, we included data from the T2-T5 group. When analysing the relationship between T3 tilt and CA, we included data from the T3-T5 group. When analysing the relationship between T4 tilt and CA, we included data from the T4-T5 group. When analysing the relationship between T5 tilt and CA, we included data from the T5 group. This can effectively avoid the impact of surgical internal fixation on the analysis results.

**Figure 2:**
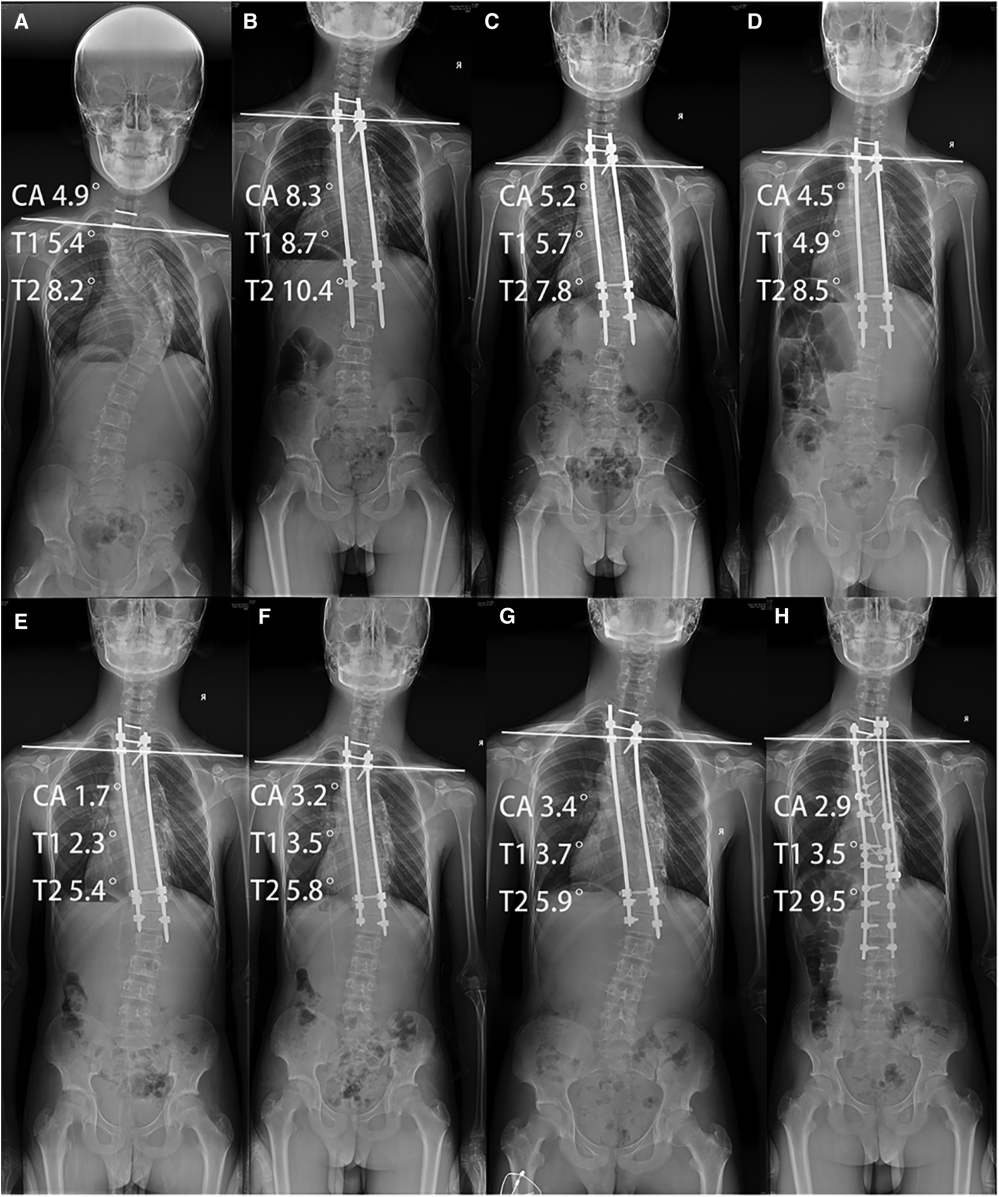
(**A**) Represent the preoperative entire spine radiograph of patients with the EOS patient, (**B**–**G**) represent entire spine radiograph after each growth rod extension surgery, and (**H**) represent the entire spine radiograph after the last growth rod extension and spinal fusion fixation surgery.

When analyzing EOS patients, we only included data of postoperative CA changes and T1–T5 tilt angle changes, and analyzed their correlation, as shown in Figure 2. Although there were only 10 EOS patients, due to the need for multiple surgeries using growth rod technology, each patient had multiple changes in T-tilt and CA after surgery. We ultimately obtained 60 data of changes in T-tilt and CA after corrective surgery. In addition, the data of EOS patients were obtained from multiple surgeries of the same patient, which is of great significance. We divided patients with the UIV located in T1–5 into five groups: Group T1, Group T2, Group T3, Group T4 and Group T5. To avoid the impact of surgical internal fixation on the inclination angle of the vertebral body, we only measured the tilt angle at and above the UIV when conducting data statistics. For example, when the UIV is located at T2, we only measure the tilt angle of T1 and T2. So, the data volume of T2 is the total data volume minus the data with UIV located at T1.

### 2.2 Statistical analysis

All parameters were expressed as the mean ± standard deviation. Spearman's correlation coefficient was used for correlation analysis, and simple linear regression was performed simultaneously. All statistical analyses were conducted using SPSS software (version 26; IBM Corp., Armonk, NY, USA). A *p* value < 0.05 was considered statistically significant.

## 3 Results

A total of 100 AIS patients and were included in this retrospective study with mean ages of 14.02 ± 2.13 years. The majority of patients were female (78%). The demographics of scoliosis patients and measured or calculated parameters are illustrated in Table 1.

**Table 1:** Demographic data and measured and calculated parameters for patients with AIS.

Variable	T1	T2	T3	T4	T5	CA
UIV number^a^	10	25	12	18	35	
Data volume^a^	100	90	65	53	35	
Pre tilt	0.91 ± 4.67	−1.10 ± 6.55	−7.56 ± 8.55	13.31 ± 9.53	−16.66 ± 11.99	−1.56 ± 3.02
Post tilt	3.44 ± 3.82	2.50 ± 4.52	−1.25 ± 5.55	−3.76 ± 5.92	−7.03 ± 6.77	0.88 ± 2.10
(Post-Pre) change	2.52 ± 3.36	3.52 ± 4.86	5.97 ± 6.88	9.09 ± 8.14	9.63 ± 9.56	2.48 ± 3.36

^a^ We divided patients with the UIV located in T1–5 into five groups: Group T1, Group T2, Group T3, Group T4 and Group T5. To avoid the impact of surgical internal fixation on the inclination angle of the vertebral body, we only measured the tilt angle at and above the UIV when conducting data statistics. For example, when the UIV is located at T2, we only measure the tilt angle of T1 and T2. So the data volume of T2 is the total data volume minus the data with UIV located at T1. CA, clavicle angle; T, thoracic vertebra; UIV, upper instrumented vertebra; Pre, preoperative; Post, postoperative.

A total of 10 EOS patients and were included in this retrospective study with mean ages of 9.69 ± 1.8 years. The majority of patients were female (70%). The demographics of EOS patients and measured or calculated parameters are illustrated in Table 2.

**Table 2:** Demographic data and measured and calculated parameters for patients with EOS.

Variable	T1	T2	T3	T4	T5	CA
UIV number^a^	0	2	2	4	2	
Data volume^a^	60	60	48	36	12	
Pre tilt	−2.62 ± 12.22	−3.34 ± 15.64	−10.65 ± 13.85	2.41 ± 18.31	−40.60 ± 0	−1.13 ± 4.94
Post tilt	−2.60 ± 11.19	−2.45 ± 13.37	−7.20 ± 8.66	−4.46 ± 9.31	−23.57 ± 1.36	0.29 ± 3.77
(Post-Pre) change	−0.86 ± 3.50	−0.72 ± 4.85	1.50 ± 9.87	−7.08 ± 10.00	16.70 ± 0.78	−0.76 ± 3.36

^a^ Due to the need for multiple surgeries using growth rod technology, each EOS patient had multiple changes in T-tilt and CA after surgery. We ultimately obtained 60 data of changes in T-tilt and CA after corrective surgery. We divided patients with the UIV located in T1–5 into five groups: Group T1, Group T2, Group T3, Group T4 and Group T5. To avoid the impact of surgical internal fixation on the inclination angle of the vertebral body, we only measured the tilt angle at and above the UIV when conducting data statistics. For example, when the UIV is located at T2, we only measure the tilt angle of T1 and T2. So, the data volume of T2 is the total data volume minus the data with UIV located at T1. CA, clavicle angle; T, thoracic vertebra; UIV, upper instrumented vertebra; Pre, preoperative; Post, postoperative.

The correlation between preoperative CA and T1–T5 inclination angles, postoperative CA and T1–T5 inclination angles, and the correlation between the change in T1–T5 inclination angles after corrective surgery and the change in CA were analysed and presented in the following tables.

**Figure 3:**
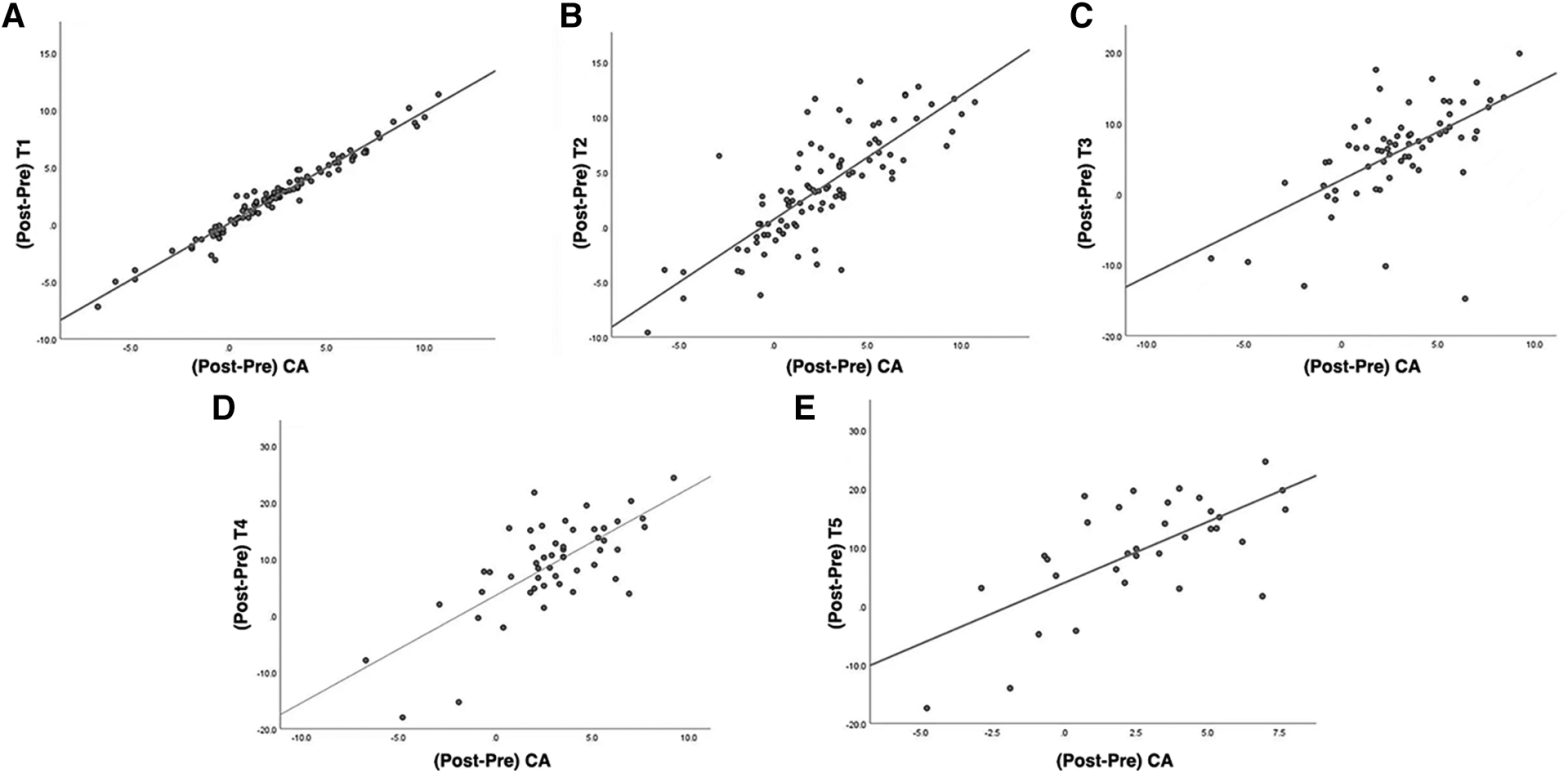
(**A**–**E**) Represent scatter plots and linear regression relationships between changes in T1-5 tilt angle and CA after corrective surgery in T1, T2, T3, T4, and T5 groups, respectively.

**Figure 4:**
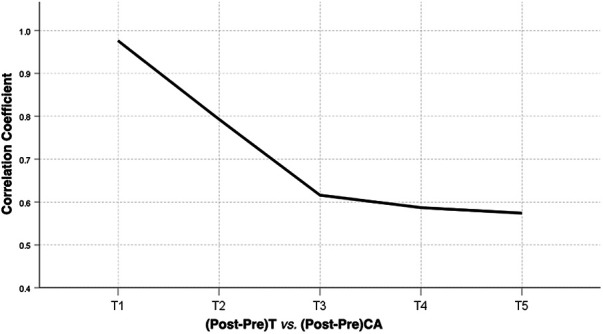
A line graph of correlation coefficient between T1−T5 tilt angle change and CA change before and after correction surgery of AIS patients. CA, clavicle angle; T, thoracic vertebra; Pre, preoperative; Post, postoperative.

**Figure 5:**
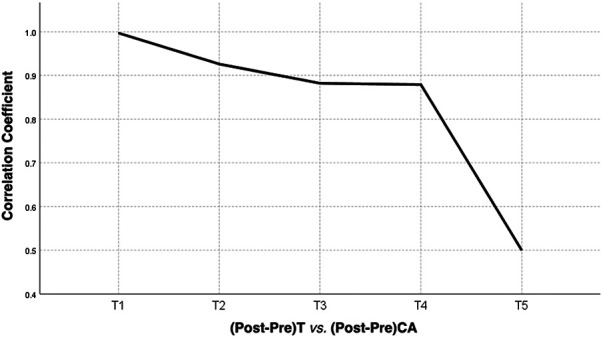
A line graph of correlation coefficient between T1−T5 tilt angle change and CA change before and after correction surgery of EOS patients. CA, clavicle angle; T, thoracic vertebra; Pre, preoperative; Post, postoperative.

Statistical analysis showed that there was a correlation between the preoperative T1 tilt angle and preoperative CA and between the postoperative T1 tilt angle and CA, with correlation coefficients of 0.639 (*P* < 0.01) and 0.506 (*P* < 0.01), respectively. The change in T1 tilt angle after corrective surgery was significantly correlated with the change in CA, with a correlation coefficient of 0.976 (*P* < 0.01) (correlation coefficients and *p* values are shown in Tables 3–5). There was a correlation between the preoperative T2 tilt angle and preoperative CA and between the postoperative T2 tilt angle and CA, with correlation coefficients of 0.614 (*P* < 0.01) and 0.374 (*P* < 0.01), respectively. The correlation coefficient between the change in T2 after corrective surgery and the change in CA was 0.793 (*P* < 0.01), indicating a significant correlation. The correlation coefficients between the preoperative T3 tilt angle and preoperative CA and between the postoperative T3 tilt angle and CA were 0.430 (*P* < 0.01) and 0.300 (*P* < 0.05), respectively. The correlation coefficient between the change in T3 after corrective surgery and the change in CA was 0.616 (*P* < 0.01). The correlation coefficients between the preoperative T4 tilt angle and preoperative CA and between the postoperative T4 tilt angle and CA were 0.495 (*P* < 0.01) and 0.312 (*P* < 0.05), respectively. The correlation coefficient between the change in T4 after corrective surgery and the change in CA was 0.587 (*P* < 0.01). The correlation coefficient between the preoperative T5 tilt angle and preoperative CA was 0.526 (*P* < 0.05), while there was no correlation between the postoperative T5 tilt angle and postoperative CA. The correlation coefficient between the change in T5 after corrective surgery and the change in CA was 0.574 (*P* < 0.01). The results of the linear regression analysis and a line graph of correlation coefficient are displayed in Figures 3, 4, respectively. As a supplement to the data, EOS patients showed a significant correlation between changes in T1 tilt angle and changes in CA after corrective surgery, with a correlation coefficient of 0.997 (*P* < 0.01) (the correlation coefficient and *P*-value are shown in Table 6). By analysing these data, we found that as the vertebrae moved downwards, the correlation coefficient gradually decreased, as shown in Figure 5.

**Table 3:** Correlation coefficient analysis of preoperative CA and T1–T5 inclination angles of AIS patients.

Variables	Correlation coefﬁcient	*p* value
Pre-T1 vs. Pre-CA	0.639**	<0.001
Pre-T2 vs. Pre-CA	0.614**	<0.001
Pre-T3 vs. Pre-CA	0.430**	<0.001
Pre-T4 vs. Pre-CA	0.495**	<0.001
Pre-T5 vs. Pre-CA	0.526*	0.002

CA, clavicle angle; T, thoracic vertebra; Pre, preoperative; Post, postoperative.

* *p* < 0.05.

** *p* < 0.01.

**Table 4:** Correlation coefficient analysis of postoperative CA and T1–T5 inclination angles of AIS patients.

Variables	Correlation coefﬁcient	*p* value
Post-T1 vs. Post-CA	0.506**	<0.001
Post-T2 vs. Post-CA	0.374**	<0.001
Post-T3 vs. Post-CA	0.300*	0.015
Post-T4 vs. Post-CA	0.312*	0.025
Post-T5 vs. Post-CA	0.272	0.125

CA, clavicle angle; T, thoracic vertebra; Pre, preoperative; Post, postoperative.

* *p* < 0.05.

** *p* < 0.01.

**Table 5:** Correlation coefficient analysis of the change in CA and the change in T1–T5 inclination angles of AIS patients after corrective surgery.

Variables	Correlation coefﬁcient	*p* value
(Post-Pre) T1 vs. (Post-Pr9e) CA	0.976**	<0.001
(Post-Pre) T2 vs. (Post-Pre) CA	0.793**	<0.001
(Post-Pre) T3 vs. (Post-Pre) CA	0.616**	<0.001
(Post-Pre) T4 vs. (Post-Pre) CA	0.587**	<0.001
(Post-Pre) T5 vs. (Post-Pre) CA	0.574**	<0.001

CA, clavicle angle; T, thoracic vertebra; Pre, preoperative; Post, postoperative.

** *p* < 0.01.

**Table 6:** Correlation coefficient analysis of the change in CA and the change in T1–T5 inclination angles of EOS patients after corrective surgery.

Variables	Correlation coefﬁcient	*p* value
(Post-Pre) T1 vs. (Post-Pre) CA	0.997**	<0.001
(Post-Pre) T2 vs. (Post-Pre) CA	0.926**	<0.001
(Post-Pre) T3 vs. (Post-Pre) CA	0.882**	<0.001
(Post-Pre) T4 vs. (Post-Pre) CA	0.879**	<0.001
(Post-Pre) T5 vs. (Post-Pre) CA	0.500**	0.667

CA, clavicle angle; T, thoracic vertebra; Pre, preoperative; Post, postoperative.

** *p* < 0.01.

## 4 Discussion

Adolescent idiopathic scoliosis is the most common spinal deformity and has a high diagnostic relevance in the pediatric population, mostly during adolescence (12). The Scoliosis Research Society (SRS) suggests bracing for patients with a Cobb's angle greater than 25°. Surgery is indicated for subjects with a Cobb's angle greater than 45°-50° (13). For severe EOS patients, surgery is the only effective method, growing rods are now considered the gold standard thanks to the work of Akbarnia and others (3, 4). Regardless of the surgical method, postoperative shoulder imbalance is a significant complication.

Shoulder imbalance (SI) after spinal scoliosis correction surgery is regarded as a critical problem. It not only affects the appearance of patients but also leads to many long-term complications, such as the distal adding-on phenomenon (14). Achieving shoulder balance after spinal scoliosis correction surgery is the goal of many surgeons and is also pursued by patients and their families. Shallu Sharma once pointed out in his research that in addition to influencing clinical and functional outcomes, trunk/shoulder deformity contributes substantially to negative self-image, psychological distress and concerns over body development (15). There have been many studies on shoulder imbalance after spinal scoliosis correction surgery, such as studies that determined the appropriate T1 tilt angle (14, 16, 17), studies that determined the appropriate correction of the MT curve (18, 19), studies that evaluated the corresponding measures for different preoperative shoulder level differences (20) and studies that evaluated the selection of the UIV for upper fixation vertebra (18, 21, 22). Dr Zhang summarized past research and drew conclusions that despite the plentiful research on this topic, this complication still remains prevalent in patients with spinal scoliosis surgical patients, with a combined incidence rate of 25% for PSI (5).

Shoulder imbalance can be classified into two different types: medial shoulder imbalance (MSI) and lateral shoulder imbalance (LSI) (6). Current studies have shown that patients with AIS after spinal scoliosis correction surgery attach more importance to LSI because LSI has a greater impact on appearance compared to MSI (23). MSI is reflected radiologically by the T1 tilt angle (the angle between the horizontal line and the line passing through the endplate of T1), the first rib angle (FRA, the angle of inclination between the tangents that connect the two tops of the first rib), and the degree of curvature at the proximal thoracic (PT) region (7, 8). Lateral shoulder imbalance (LSI) is reflected by the clavicle angle (CA, the angle between the highest point of each clavicle and the horizontal line) and the difference in the heights of the manubrium (CHD, measured by drawing a horizontal line at the upper edge of each manubrium) (8, 9). CA is closely related to LSI (5, 24). The T1 tilt angle has a better correlation with MSI but a poor correlation with LSI and cannot serve as a substitute indicator for lateral shoulder balance (LSB) (10). However, our study found that even if the T1 tilt angle has a poor correlation with the CA, the change in the T1 tilt angle has a close correlation with the change in the CA and plays a great role in the LSI after corrective surgery. Description of shoulder anatomy in the literature shows that there is no direct contact between the spine and the shoulder. The spine vertebra articulates with the ribs, which then loosely connect with the scapula (25, 26). Through the costotransverse joint, costal head joint, sternocostal joint, and sternoclavicular joint, the first rib connects the T1 vertebrae and the clavicle, which are also reinforced by the anterior sternoclavicular ligament and costoclavicular ligament. Therefore, the T1 vertebrae, the first rib and the clavicle can be regarded as a complex that has a linkage function. T1 can adjust the clavicle, the angle of which (CA) is closely related to LSB, through the first rib. Although previous literature reported that T1 tilt had a poor correlation with clinical shoulder appearance (9), we found that the changes in T1 tilt before and after correction surgery were significantly related to the changes in CA through a retrospective analysis of previous AIS cases, which indicates the possibility of this complex topic and may provide a practical, digitally controllable method for controlling postoperative LSB.

This study used a retrospective analysis to investigate the relationship between T1–T5 tilt angles and the clavicle angle (CA) and found that the change in T1 tilt angle after orthopaedic surgery of AIS patients was significantly correlated with the change in CA, with a correlation coefficient of 0.976, indicating a significant relationship between T1 and the clavicle. The correlation coefficients between the change in T2, T3, T4, and T5 after corrective surgery and the change in CA were 0.793, 0.616, 0.587, and 0.574, respectively. The correlation coefficients between changes in T1, T2, T3, and T4 after corrective surgery and changes in CA in EOS patients were 0.997, 0.926, 0.882, and 0.879, respectively and there was no correlation between the changes in T5 tilt angle and changes in CA. From these data, we found that the correlation gradually decreased as the vertebrae moved down, which may be related to compensation in the intervertebral disk. In previous research, we found a correlation between T1 changes and CA changes, took into account the flexibility between T1 and UIV, and proposed a formula to predict the ideal postoperative UIV tilt to prevent lateral shoulder imbalance after scoliosis correction surgery (27). However, in that study, we only found the phenomenon and did not conduct any further study. In the current study, we analysed the correlation between preoperative T1, T2, T3, T4, and T5 tilt angles and preoperative CA and postoperative T1, T2, T3, T4, and T5 tilt angles and postoperative CA and found that the correlation coefficients were obviously smaller than the correlation coefficient between the change in T1, T2, T3, T4, and T5 after corrective surgery and the change in CA. By comparing the correlation coefficients, it was found that there may be no strong correlation between T1–T5 tilt and CA before and after the operation, but the correlation coefficient of their changes increased. For example, the correlation coefficients between preoperative T3 tilt angle and preoperative CA and postoperative T3 tilt angle and CA were 0.430 and 0.300, respectively, while the correlation coefficient between the change in T3 after corrective surgery and the change in CA was increased to 0.616. Therefore, when treating patients with a UIV between T1–T5, we should pay special attention to the changes in the UIV angle before and after scoliosis correction surgery. In addition, our previous clinical work has already applied the research by predicting the appropriate tilt angle of the UIV through a certain formula to achieve postoperative shoulder balance as much as possible. In our previous study, we described the test method in detail, and in a prospective study, 13 scoliosis patients all achieved a satisfactory LSB (27). On the other hand, the results of the current study complement the previous data and make the previous articles seem more logical.

This study has some limitations. First, this observational study involved retrospective data collection, which may have resulted in some measurement delta. Second, due to the limited number of cases in this preliminary clinical study, the conclusions need further validation. After this preliminary report, larger sample studies should be conducted. Despite these limitations, to the best of our knowledge, this is the first retrospective study to investigate the relationship between T1–T5 tilt angles and CA.

## 5 Conclusions

In summary, this study suggests that there is a close relationship between T1–T5 and the clavicle and that the change in T1 tilt angle after spinal scoliosis correction surgery is significantly correlated with CA, which decreases as the vertebra moves down. This also highlights the need to pay attention to changes in the tilt angle when dealing with UIV selection for patients at T1–T5. Achieving shoulder balance after orthopaedic surgery is of great significance for patients with scoliosis.

## Data Availability

The original contributions presented in the study are included in the article/Supplementary Material, further inquiries can be directed to the corresponding authors.
